# A Dissertation on the Diseases of the Maxillary Sinuses

**Published:** 1842-06

**Authors:** W. W. H. Thackston

**Affiliations:** candidate for the degree of Doctor of Dental Surgery.


### ARTICLE II.

*A Dissertation on the Diseases of the Maxillary Sinuses.* Presented to the Faculty of the Baltimore College of Dental Surgery, by W. W. H. Thackston, candidate for the degree of Doctor of Dental Surgery. Baltimore, February 14, 1842.

In treating upon the diseases of the *Antra Highmorianum*, it may not be improper to examine the relation which these cavities sustain to the surrounding organs. To this end I deem it unnecessary to go into a minute detail of the anatomy of the parts, but will content myself, with merely noticing the most prominent and important features, as they present themselves.

They (the maxillary sinuses) are found in each superior maxillary bone, the palatine and alveolar processes forming their floors, the orbiter processes constituting their roofs, the cheeks their external, and the nasal plate of the superior maxilla their internal parietes. In the nasal plate is found a hole, through which the mucous secretion of the antrum is discharged; these sinuses are lined by a continuation of the schneiderian or pituitary membrane of the nose. There is then between them, the nose, the organ in which the sense of smell is located, and through which the process of respiration is principally carried on, immediately above them, the organs of vision; and below them, those of mastication. From this hasty glance at the important organs, contiguous to the antral sinuses, the importance of the various diseases to which they are subject may be appreciated, and the injury that may result from them, to the parts, above mentioned. I do not at the present time propose taking up all the diseases incident to these cavities, but only to examine in detail those which are most frequently met with. I shall however at the same time take a hasty glance at some of those forms of disease, which occasionally develope themselves in one or both *antra*, and which are dependent upon a specific constitutional diathesis.

The various forms of *carcinoma* as *fungus hœmatodes, &c.* belong to this class, and by a reference to the pathology of these affections it would seem that after their full development, (and it is rarely till then that they attract attention) it is seldom that any advantage results from interference with them. In the first or almost inceptive stage, when the local affection consists of nothing more than a small circumscribed tumour, its total eradication, together with all the contiguous parts which may be at all affected, will sometimes cure the disease and afford the patient an opportunity of dying of some less loathsome and horrible malady. But the same tendency which at first determines its development, still exists, and the constitutional vices upon which it is dependent often lurk too securely in the system to be reached or eradicated by any means with which the art of medicine is at present possessed. In a large majority of instances, therefore, those who are so unfortunate as to possess constitutional vices of this kind must be resigned to the melancholy fate which awaits them,—experience having demonstrated, that after the full development of a *fungus hœmatodes*, or carcinomatous ulcer, though by active and persevering treatment its progress be arrested in one place, it soon makes its appearance in another.

Of the diseases which more particularly claim my attention on the present occasion; I shall first notice *Muco-Purulent Secretion,* or as some have denominated it, dropsy, and others, abscess of the antrum. This is by far, the most common affection to which the maxillary sinuses are liable. This disease consists of nothing more than the name implies, a vitiated or altered state of the mucous secretions. It will be readily perceived from the propinquity of the parts and the continuity of structure which exists, that most causes tending to induce a morbid action in the mucous tissues of the mouth and nose, may readily extend their effects to the parts now under consideration. Thus, the irritation produced by common catarrh, may, and does very frequently extend itself to the antrum, causing a thickening of the lining membrane, so much so as occasionally to obliterate the nasal opening; the increased secretion consequent upon the inflamed or irritated membrane thus becomes pent-up, vitiated, and in turn, the exciting cause of a high state of inflammation, which not unfrequently results in the suppuration of some of the mucous follicles, and sometimes in the destruction of some part of the membrane, when a necrosis and perhaps exfoliation of the subjacent bone ensue. In the meantime the accumulation of purulent matter increases, until the cavities no longer capable of containing it, are enlarged or broken down by the yielding of some part of their bony parietes. I am, however, induced to believe, that this affection more frequently results from the irritation produced by the presence of diseased or dead teeth than from any other cause.

In the first place it is not perhaps in more than one case out of twenty that it is met with unattended by a diseased state of the subjacent teeth, and in few instances I believe, would any other treatment than the restoration or removal of these organs, be required to effect a cure. Another circumstance which goes strongly to sustain the views which I have taken, as to the most common cause of this affection, is the very great difference in the liability of the frontal and maxillary sinuses to diseases. These cavities are analagous; they are lined by the same membrane, and apart from the absence of diseased teeth. I can conceive of no circumstances which could render the former less liable to disease than the latter, unless it be that the outlet for the secretion is more favourable to its discharge in one instance than in the other, but this may be considered as questionable. But if it is admitted, the fact that irritation and inflammation precede all other diseased actions in these parts, and if these cause an obliteration of the nasal openings of the maxillary sinuses, would they not also be likely to cause the openings into the frontal to close, and their secretions become vitiated and as acrid as those of the antrum? Yet this rarely happens, while on the other hand, so common is the affection of which I am treating, that few in the profession, have not had opportunities of meeting with it.

It is asserted by some, that the fangs of the first and second superior molares penetrate the sinuses; but this is denied by others; that is, in the natural state of the parts. As to the various opinions entertained upon this subject, it is not necessary for me at this time speak: of one fact however, I am very certain, and that is, in no instance, does anything more than a thin spongy layer of bone intervene; through which a diseased action may be readily communicated, from the fangs of the subjacent teeth, to the membranous tissues of the antrum. As to the manner in which the fangs of the molares, are made to perforate the antral cavities, (as they frequently do) whether it be natural, or the result of diseased action: I shall for the present, leave for others to determine.

The symptoms of muco-purulent secretion, when produced by catarrh, are generally slight temporary uneasiness, perhaps pain, of a dull heavy character, which readily passes off upon the application of a few leeches to the cheek, followed by anodyne fomentations, or the injection of some bland fluid into the sinus, through the nasal opening, if practicable. For this purpose tincture of myrrh diluted with tepid water, will, in most instances, be all that is necessary. But where the disease evidences a disposition to take on a more aggravated form, the dull heavy pain increases, occasionally lancinating paroxysms shoot from the maxillary tuberosity to the upper and interior part of the nose, the pulse is hard and hurried, the bowels constipated, the cheek becomes turgid, sometimes spots or blotches of a florid colour appear, great pain is experienced on pressing the parts, and unless the disease be speedily arrested, yielding of the bony parietes will ensue, or perhaps necrosis from the death of the periosteal tissue; from which the osseous tissue derives its support. Where the nasal duct remains pervious, the symptoms may vary considerably from those just described, the disease may remain for a considerable time without any other perceptible change than an increase or diminution of the discharge. In debilitated or strumous constitutions these latter symptoms not unfrequently result in the necrosis, and subsequent exfoliation of parts of the bony parietes of the sinus. Where an enlargement of the cavity or yielding of the walls occur, it is most frequently in the direction of the nose, closing, not unfrequently the nostril of the diseased side. Where a yielding of the alveolar and palatine processes occur, the gums become spongy, turgid, and ulcerated, the teeth elongated and displaced, and unless a perforation be made and the contents of the antrum evacuated, an exfoliation, with perhaps sloughing of the soft parts will soon ensue. If the nasal opening should not become pervious, and the health of the parts restored, the opening formed by the disease, or the operator, as the case may be, will become fistulous.

The treatment of this affection in its first or inflammatory stage will consist in the removal of all sources of irritation. To accomplish this, the mouth should be carefully examined; and all fangs, and teeth incapable of perfect restoration to health, should at once be removed, as should also all accumulations of a morbid character, from the healthy ones. The cheeks should then be leeched and fomented, and if practicable, some bland fluid should be injected through the nasal duct; the bowels at the same time should be opened by saline purgatives.

If the disease still continues unabated, the accumulation and situation of the secretion go on, and the nasal opening closes, a somewhat different course of treatment will be indicated. An opening into the diseased sinus, should first be formed. It not unfrequently happens that on removing the diseased teeth and fangs, which are almost uniformly met with in this affection, a communication is found to have been established between the socket of some one of their fangs and the antrum, this communication if not of a sufficient size, should be enlarged. Where there are no diseased teeth or fangs, a healthy molar tooth must be sacrificed; the second is the one usually selected, as it is situated under the most dependent part of the *sinus* and is very accessible to the operator. The perforation, *ceterus paribus,* should be made through the anterior external alveolus, but it may be made through either of the alveolar cavities of the fangs of the first or second molares, the bicuspides, or cuspidati, though a much thicker table of bone will have to be penetrated when the perforation is made through an alveolus of any of the other teeth.

From the difficulty of access, and the peculiar situation of the *dens sapientiæ,* it would be obviously improper to attempt the operation through its socket.

The manner of performing the above-mentioned operation, is simply to introduce a trocar into the alveolus selected, and with the hand properly guarded, to prevent injury to any of the parts, by the sudden or unexpected entrance of the instrument into the sinus, gently rotate it, until the cavity is reached, which in most instances will be very soon, as the intervening osseous layer is not only thin, but generally spongy and soft. On reaching the cavity and withdrawing the instrument, a gush of matter ensues, generally of a thick, fetid character, though in this respect it is variable; the state or quality of the pus depends entirely upon the changes which have been effected by the disease. Where the operation has been early performed, the discharge may only be thickened, clouded, and nearly inodorous, but when it has been deferred, the matter long confined, and portions of the lining membrane destroyed, attended with caries or necrosis of the bony parietes, the matter will be found to have assumed a thick, viscid, and intolerably fetid character. As there is always considerable irritation produced in the parts by the operation, it will be proper to open the bowels with gentle aperients, and to apply a few leeches to the cheek, or take a few ounces of blood from the arm. The sinus should at first be injected with some mild, bland fluid, as tepid water, or tincture of myrrh, diluted with tepid water or sweet milk. The strength of the injections should be gradually increased, being at the same time careful to avoid having them strong enough to light up inflammation in the lining membrane; yet they should be always sufficiently strong to stimulate the parts; and Mr. Bell says, "to leave a sensation of heat an hour or two after their employment."

The remedial agents used in this affection are numerous and various, the most efficient of which are tincture of myrrh, port wine diluted, or otherwise, solution of the sulphate of zinc, in the proportions of from two to eight grains to two ounces of water; argent. nitratum, one grain to two or three ounces of water ; these may be strengthened or diluted as occasion may require.

The treatment which we have detailed, if persisted in, if the system be favourable to the local treatment, (and this should never be lost sight of, for the successful treatment of all local affections depends materially upon it,) will generally establish a healthy action in the maxillary sinuses, if they be not affected with any other disease, than muco-purulent secretion, or as it is more frequently denominated, abscess. It may here be observed, that the nasal duct almost always becomes pervious after the membrane regains its healthy action, the artificial perforation should then be permitted to close, and not before; and to prevent which, it should be kept open by the introduction of a wax bougie, or some similar article prepared for the purpose. Should the artificial perforation become fistulous, the removal of its inner wall, in the form of a tube, will rarely fail of effecting the object desired.

I will here take occasion to advert to a circumstance, which I should have noticed before. In muco-purulent secretion of the antrum, there exists sometimes, white, opaque, curdy flocculi, in such quantity as to obstruct the nasal passage; and even the artificial opening through the floor of the sinus is sometimes blocked up by them. They are easily removed by injections, and cease to be formed as the membrane is restored to health.

The next affection of these cavities upon which I propose to treat, is often connected with, or grows out of the disease of which I have just spoken.

*Ulcers of the Antrum.*—These are of two classes, the first has its seat in the mucous membrane of the sinus, and may be attributed to the following causes, viz: the presence of extraneous bodies, the accumulation and retention of vitiated secretions, the irritation produced by the presence of diseased teeth or fangs, the formation of alveolar abscesses, exostosis of the fangs of the teeth; or it may occur as an idiopathic disease of the lining membrane, induced by a scorbutic tendency, or venereal taint of the system. It may be properly observed here, that the particular form or character which these diseases assume, is dependent upon the tendency, or diathesis of the system; therefore, causes which in one instance would only produce a vitiated secretion, would in others, give rise to polypi, osteo sarcoma, or fungus hæmatodes. It is then obvious that very many diseases, differing essentially in their character, and therapeutical indications, may and do originate from the same causes, and it is important that this fact should be borne in mind.

The symptoms which characterize the affection, of which we are now treating, are continued pain in the antrum, with a discharge of slightly fetid pus, this evacuates itself at first through the nasal duct, but soon finds its way through numerous fistulous openings formed in the floor of the sinus. These ulcers sometimes take on a fungoid character; and fill up the sinus. In this state they are often mistaken for polypi: we can however, distinguish these tumours from polypus, by the bloody, fetid sanies which is discharged.

The treatment of this disease is, in its first stage, similar to that recommended in muco-purulent secretion, consisting in the removal of all sources of irritation, the evacuation of the morbid contents of the sinus, and the employment of stimulating and astringent injections. Where the affection takes on the fungoid character which we have mentioned, the sinus should be laid open, and the whole of the diseased mass removed with the knife and cautery. Should a reproduction of the tumour be indicated, the parts should be touched with the actual cautery, or the arsenical solution recommended by Mr. Bell, may with advantage be applied. After the extirpation of the diseased mass, the health of the parts may be restored by the use of astringent and stimulating injections, as the solution of the sulphate of zinc, tincture of myrrh, solution of argent. nitratum, &c.

The second class of ulcers found in the antrum are characterized by disease or death of the subjacent bone, and it may here be observed that the diseases of the bones are in many respects analagous to those of the soft parts, both being liable to irritation and inflammation, which they reciprocally communicate to each other. The bones, from their being less liberally endowed with nerves, and blood-vessels are more readily deprived of vitality than the more highly organized tissues. The disease now under consideration may grow out of simple ulcer, or any cause which will light up inflammation in the periosteal tissue, as the presence of diseased or dead teeth, the excessive use of mercury, a venereal taint, scorbutic diathesis, &c.

The symptoms are pain, a blackish and intolerably fetid discharge, where the disease is the result of a scorbutic tendency, it may be distinguished by the general scorbutic indications, as a leaden, ashy hue, sponginess and turgidity of the gums, ulcers upon the inferior extremities, &c. where it is caused by venereal taint, it is known from the previous history of the disease, and the nocturnal pains which characterize all affections growing out of this cause.

The proper course of treatment, will be to make an opening into the antrum sufficiently large for the evacuation of the diseased matter, and the removal of any dead pieces of bone, which may be found. As the affected bone cannot be cured, our object will be, to arrest the necrosis, and to aid nature in the exfoliation of such parts, as soon as they are entirely detached from the living bone. The *sequestra,* however, should not be interfered with, until they are entirely detached, for by so doing we might inflict more injury, than their presence would occasion. This together with such other local treatment, as the symptoms may indicate, will generally effect a cure if the cause be local; if however, it should result from any of the constitutional causes which have been mentioned, of course anti-scorbutic, venereal, or mercurial treatment should be adopted.

*Exostosis of the Antrum.*—This disease as are most others of the antrum, is much oftener than otherwise, produced by the irritation consequent upon the presence of diseased dental organs, it may however, occur independently of this cause; the law, that the particular development of these affections, is dependent upon the tendency or diathesis of the general system, will apply to this, as well as other forms of disease.

The symptoms are, a nutritive action of the fibrous coat of the mucous membrane or periosteum of the antrum, the subjacent bone becomes thickened by continual ossific matter, the membrane also becomes thickened and in some instances the whole cavity becomes nearly or quite filled; if suffered to progress, the destruction of the contiguous parts will ensue, and the ultimate termination of course be fatal. This kind of tumour may be distinguished by its heaviness or the sense of weight which the patient experiences, gives but little pain at first and is consequently not soon noticed. When it attains to considerable size the pressure upon the walls of the sinus will of course produce more or less pain, as the pressure may be slight or considerable.

The treatment will consist in the removal of the whole of the diseased mass, and the destruction of any part of the membrane by the cautery, which evidences a diseased tendency. This operation, will be subsequently described. The parts may then be restored to health by the same course of treatment advised for muco-purulent secretion. I will now pass on to the consideration of a species of tumour sometimes found in the antrum, denominated.

*Osteo Sarcoma.*—This, like the foregoing disease, results from a morbid action of the periosteal tissue, and I would here observe that all morbid growths arising from the periosteal tissue, are of a fibrous character, while those having their origin in the mucous tissue are vascular. This fact will enable us always to determine upon the origin, of those enlargements, and consequently the course of treatment most proper to adopt. Osteo sarcoma of the antrum, is a fibrous tumour, interspersed with patches of bony matter, the proportion of osseous matter however varies, existing in much larger quantities in some of these tumours than others. They grow rapidly, produce pain varying from that of a dull heavy character, to the most lancinating paroxysms, they suppurate, destroy the contiguous organs, and with them life.

The treatment to be available, must be prompt and decisive, for nothing short of an operation for the removal of the tumour will be productive of the least benefit; this to be effectual should be done in the early stage of the disease; for if deferred for a length of time, so debilitated and emaciated does the patient become, that there is great danger of his dying under the operation, therefore, under such circumstances, it would never be advisable to attempt its performance.

When an operation is determined upon, we are to prepare ourselves with suitable instruments. Those recommended by Professor Gibson, of the Pennsylvania University, consist of chisels, gouges, mallets, &c., but as the bony walls of the sinus are thin, and often very much softened by the disease, instruments less formidable should be preferred. The teeth or fangs, if there be any beneath the part to be operated upon, should be extracted, the gum should then be dissected up, and the external and inferior walls of the sinus removed with a sharp and strong pointed knife, cutting forceps, &c. The opening should in all instances be made sufficiently large for the ready removal of the whole tumour which in most cases may be soon accomplished by the use of properly shaped knives, and excising forceps, for separating adherent pieces of bone.

After the suppression of the hemorrhage, which is often profuse, the dressings should be removed, and the parts carefully examined, and any portions of doubtful appearance should be removed or destroyed by the actual cautery.

Should a reproduction of the tumour be indicated, the application of a moderately strong solution of arsenic, so managed as not to escape into the mouth, which may be accomplished in the following manner, will suppress it. Introduce a sponge of proper size, saturated with the solution into the antrum, then pass into the mouth, a semi-lunar shape piece of oiled silk, leaving its outer edge projecting out of the mouth; this should be adjusted and retained in its proper place by the patient: the solution will then escape over the silk into a basin or other receptacle, and the saliva into the same vessel from under the silk.* It need scarcely be observed, that the greatest precaution is necessary in the employment of this article.

The cure of the disease will be greatly facilitated by the employment of such injections, as the symptoms may indicate, little benefit will result from local treatment, or comparatively little, if the state of the system be not likewise attended to. As I before observed, in this affection, the system is generally found to be

* See Bell on the Teeth, p. 284.

greatly debilitated, and emaciated with suffering. To sustain and invigorate it, the use of tonics, generous diet, and if practicable, gentle exercise should be resorted to. Should febrile symptoms manifest themselves, they should be subdued by anti-phlogistic treatment. I will now take up the class of tumours denominated

*Polypi of the Antrum.*—Tumours of this class do not differ materially from polypi of the nose, they are however, generally much softer. They may and sometimes do originate in the nasal fossa, and by destroying the intervening bones force themselves into the antrum, and exist there secondarily, or they may originate in the mucous membrane of the sinus itself. They do not grow from the surface of the membrane but from its internal structure, the tumour is consequently lined by the outer lamina of the membrane. They usually grow rapidly, generally producing no inconvenience until they attain sufficient size to make pressure upon the walls of the cavity, consequently we do not know of their existence in their early stage.

The causes of this affection, Mr. Liston, in his treatise on surgery, observes "cannot be satisfactorily accounted for," I am of opinion however, that it may be very plausibly attributed to a relaxed and debilitated state of the mucous membrane, the presence of extraneous matter, or sanguineous determinations to the part.

The symptoms which characterize it, are, after the tumour has attained to considerable size, dull heavy pain, sense of weight, pain increases to the most exquisite paroxysms, the gums become turgid and spongy, the teeth are loosened and displaced, ulcers form in the mouth and nose, from which is discharged a fetid sanies, some part of the bony parieties give way and the tumour is precipitated into the mouth or nasal fossa, most frequently the latter. Its true character is then, and not till then, distinctly ascertained.

The therapeutical indications in this disease are simple, consisting principally in the removal of the morbid mass as in osteo sarcoma, and the employment of stimulating and astringent local applications, with suitable constitutional treatment.

We have now briefly noticed most of the diseases incident to the maxillary sinuses; and in conclusion, I beg leave to offer a few reflections, which now suggest themselves.

The views which I entertain, and have imperfectly expressed in this thesis, I am aware are dissented from in some particulars, by those whose attainments and experience, bespeak for their opinions the most deferential respect, and I am gratified with the reflection, that those differences are comparatively unimportant, at least so far as they influence the treatment of the various disorders upon which I have treated. In ascribing the causes of a large majority of diseases that develope themselves in the antrum, to a diseased state of the dental organs, or more properly to the irritation produced by their presence, I think I do nothing more than make a deduction, sustained not only by the phenomena of the diseases in question, but by the success of treatment predicated upon the opinions which I have endeavoured to set forth.

I do not, however, wish to be considered as regarding the maxillary sinuses as susceptible of morbid impressions from diseased dental organs only, on the contrary, I wish it distinctly understood, that I believe the antrum in common with other parts of the body, to be subject to disorders totally disconnected with, and independently of the health of the teeth. But I at the same time regard the irritation arising from a diseased condition of these organs as being altogether and by far the most frequent cause; and I believe proper attention to their health and preservation, or their prompt removal when they become so much diseased as to render their restoration impracticable, will wholly avert most of the malignant forms of disease incident to the antrum maxillare.

